# Risk factors and genetic characteristics of the carriage of hypervirulent and carbapenem-resistant *Acinetobacter baumannii* among pregnant women

**DOI:** 10.3389/fmicb.2024.1351722

**Published:** 2024-03-20

**Authors:** Chao Zheng, Defeng Li, Yinglan Wang, Lisheng Wang, Yuting Huang, Jun Yao

**Affiliations:** ^1^Department of Gastroenterology, Shenzhen People’s Hospital (The Second Clinical Medical College of Jinan University, The First Affiliated Hospital of Southern University of Science and Technology), Shenzhen, China; ^2^Bacteriology and Antibacterial Resistance Surveillance Laboratory, Shenzhen Institute of Respiratory Disease, Shenzhen People’s Hospital (The Second Clinical Medical College of Jinan University, The First Affiliated Hospital of Southern University of Science and Technology), Shenzhen, China; ^3^Integrated Chinese and Western Medicine Postdoctoral Research Station, Jinan University, Guangzhou, China; ^4^School of Materials and Environmental Engineering, Shenzhen Polytechnic University, Shenzhen, China; ^5^Department of Obstetrics and Gynecology, Shenzhen People’s Hospital (The Second Clinical Medical College of Jinan University, The First Affiliated Hospital of Southern University of Science and Technology), Shenzhen, China; ^6^Department of Head and Neck Surgery, Cancer Hospital Chinese Academy of Medical Sciences Shenzhen Center, Shenzhen, China

**Keywords:** carbapenem-resistant *Acinetobacter baumannii*, high virulence, maternal carriage, genotyping, risk factors

## Abstract

**Background:**

Carbapenem-resistant *Acinetobacter baumannii* (CRAB) and its emerging evolutionary branch toward hypervirulence have been neglected in pregnancy.

**Methods:**

From September 2020 to August 2021, an active surveillance culture program encompassed 138 randomly selected pregnant women, with five subjected to sample collection at two different time points. The clinical characterization was explored through statistical analysis. Whole-genome sequencing, a *Galleria mellonella* infection model, and a global database were used to investigate the genetic characterization, pathogenicity, evolutionary history, and phylogenetic relationships of the isolates.

**Results:**

Of the 41 CRAB isolates obtained, they were divided into four Clusters^RS^ and an orphan pattern. Cluster^RS^ 1 (*n* = 31), with eight complex types in pregnancy, was also the dominant Cluster^RS^ globally, followed by Cluster^RS^ 13 (*n* = 5), identified as hypervirulent KL49 CRAB, exhibiting phylogeographical specificity to Guangdong. A maternal carriage CRAB rate of 26.09% (36/138) was revealed, with half of the isolates representing novel complex types, prominently including CT3071, as the first KL7 isolates identified in Shenzhen. Both KL49 and KL7 isolates were most commonly found in the same participant, suggesting potential intraspecific competition as a possible reason for CRAB infection without carriers during pregnancy. The independent risk factors for carriers were revealed for the first time, including advanced maternal age, gestational diabetes mellitus, and Group B *Streptococcus* infection.

**Conclusion:**

The significant carriage rate and enhanced virulence of CRAB during pregnancy emphasize the imperative for routine surveillance to forestall dissemination within this high-risk group, especially in Guangdong for Cluster^RS^ 13 isolates.

## 1 Introduction

As a pivotal pathogen of global concern, carbapenem-resistant *Acinetobacter baumannii* (CRAB) is intrinsically linked with severe, sometimes fatal, infections, prompting wide-ranging epidemiological research (Li et al., 2020; Cavallo et al., 2023; Diep et al., 2023). Efforts to understand and track the epidemiology of AB have employed a variety of genotyping methods, revealing the pathogen’s sophisticated population dynamics. This includes exploring virulence traits such as capsular polysaccharide (KL) and the lipooligosaccharide outer core (OCL), which are substantively connected to AB’s pathogenic properties (Deng et al., 2020; Li et al., 2020; Diep et al., 2023). The technological progress in whole-genome sequencing (WGS) has enriched genotyping capabilities, enabling more nuanced analysis through core genome multilocus sequence typing (cgMLST) or single nucleotide typing (SNP) for detailed typing and evolutionary studies.

An evident manifestation of CRAB’s genetic plasticity is its co-evolution toward both high virulence and drug resistance. One stark example is the KL49 AB type, similar in capsular polysaccharide type to the hypervirulent strain LAC-4 (Zhou et al., 2018; Deng et al., 2020), which has been implicated in several death-related outbreaks across continents (Valentine et al., 2008; Jones et al., 2015; Deng et al., 2020). Carbapenem-resistant hypervirulent AB (CR-hvAB) isolates with KL49 can cause fatal outcomes not only in immunodeficiency, but also in immunocompetent patients (Jones et al., 2015; Li et al., 2020). Moreover, as AB isolates, which can asymptomatically colonize various anatomical niches (Marchaim et al., 2007; Nutman et al., 2016), participate in co-infections in specific habitats, they likely engage in intraspecific competition, leveraging mechanisms such as the Type VI secretion system (T6SS) to deploy toxic effectors for direct killing of competitors (Hadjadj et al., 2018; Zhang et al., 2023).

Pregnant women who undergo physiological immunological changes, present a notable risk group for acquiring or reactivating infections (Hazenberg et al., 2021; Osei Sekyere et al., 2021), and who are carriers of AB are not only more predisposed to associated infections at an uncertain time during pregnancy, intrapartum or postpartum period, but also have a risk of maternal carriers transferring the AB to their newborns (Sood et al., 2019; Osei Sekyere et al., 2021). AB as a cause of pneumonia, urinary tract infections, and sepsis (Cavallo et al., 2023), has manifested among pregnant women and newborns in numerous countries (Sood et al., 2019; Osei Sekyere et al., 2021; Ghanchi et al., 2023; Odoyo et al., 2023; Woon et al., 2023), inciting adverse outcomes including spontaneous abortion, premature labor, and perinatal deaths (Aivazova et al., 2010; He et al., 2013; Osei Sekyere et al., 2021). Although the World Health Organization (WHO) estimates that puerperal sepsis accounts for 10.68% of maternal deaths, antepartum screening for AB remains scant, with prevailing data often emerging from retrospective studies and postpartum examinations (Say et al., 2014; Bebell et al., 2017; Osei Sekyere et al., 2021; Sousa et al., 2023).

Since the early 1970s, various reports have repeatedly emphasized the role of asymptomatic patients as a reservoir of pathogens, thus favoring their persistent colonization and extensive dissemination, and patients with positive surveillance cultures were eight times more likely to develop CRAB infections (Latibeaudiere et al., 2015), even after controlling for other variables, which emphasize the need for active surveillance for multidrug-resistant organisms and the value of molecular typing of strains to investigate their transmission and distribution features. Although there have been reported the skin or rectum being the site of higher carriage rate of CRAB (Nutman et al., 2020; Osei Sekyere et al., 2021), buccal mucosa also remains one of the most reliable sample sources to screen for its carriers (Nutman et al., 2016; Zhou et al., 2018; Cavallo et al., 2023; Da Silva et al., 2023). The oropharyngeal swabs, while may not be the best but the most viable option, could reduce sampling difficulty by healthcare professionals and enhance acceptability among pregnant women. Witnessing an upward trajectory in the dissemination and infection rates of CRAB, the objectives of this study were to assess the prevalence of oral and pharyngeal carriage of CRAB during pregnancy and investigate carriers’ high-risk factors and the genetic characteristics of related AB isolates.

## 2 Materials and methods

### 2.1 Study design and data collection

This study was part of a series of studies, including investigations into fatal outbreaks, the rapid identification of KL49 AB (Deng et al., 2020), detection of the nosocomial environment, and active surveillance culture (ASC) programs. In the present study, oropharyngeal CRAB screening was performed on pregnant women at admission (<2 h; who were required to avoid and decrease close contact with the hospitalized person before collecting swabs and over the entire hospitalization, respectively, based on the normalized prevention and control of COVID-19 epidemic) from September 2020 to August 2021 in the Department of Obstetrics and Gynecology at Longhua Branch of Shenzhen People’s Hospital. A total of 138 pregnant women (aged 20–42 years old, gestational age 5 weeks +5 days to 41 weeks) were randomly collected in the program. Among them, five pregnant women were identified as carriers of CRAB for the first time admitted, hospitalized a second time, and collected their oropharyngeal samples twice at different times during the ASC program.

Clinical data, including age, registered residence, number of pregnancy, history of vaginal delivery, cesarean section history, history of miscarriage/abortion, pre-pregnancy body mass index (BMI), number of antenatal care (ANC) visits, anemia in pregnancy, diabetes mellitus, thyroid disease, hypokalemia, hypocalcemia, hepatitis B virus (HBV) status, herpes simplex virus (HSV) status, group B *streptococcus* (GBS) infection, mycoplasma, et al. were obtained from medical records of subjects without collecting private information or disclosing information to any commercial agency. This study was approved by the Medical Ethics Committee of Shenzhen People’s Hospital (KY-LL--2019559-03). All pregnant women included in this study provided written informed consent for the use of the samples.

The database used in this study contained a total of 9,470 available AB genomes with strain-related information, including 41 CRAB genomes in the present study, 9,429 genome sequences were assembled from the National Center for Biotechnology Information (NCBI) Sequence Read Archive or downloaded from NCBI Reference Sequences (Supplementary Table 1).

### 2.2 Bacterial isolates and phenotypic characterization

In total, 41 AB isolates were collected from 36 admitted pregnant women. From 5 of these pregnant women, two isolates were collected. A total of 143 oropharyngeal samples were collected with sterile flock swabs by wiping the back wall of the pharynx with moderate force and avoiding touching the tongue, and then placed in sterile tubes for oropharyngeal swabs (Shenzhen Miraclean Technology Co., Ltd., Shenzhen, China) containing 2 mL of Luria-Bertani (LB) broth (Huankai Microbiol, Guangzhou, China). After overnight enrichment in LB broth, oropharyngeal samples were inoculated on mSuperCARBA plates (CHROMagar, Paris, France) and incubated for 24 h at 37°C for culture. Species identification was performed with the VITEK-2 compact system (bioMérieux, Marcy-l’Étoile, France) with GN ID card (bioMérieux) and confirmed by matrix assisted laser desorption/ionization-time of flight mass spectrometry (MALDI-TOF MS, bioMérieux, Marcy-l’Étoile, France) with the VITEK MS system (RUO mode). The minimal inhibitor concentrations (MICs) of ceftazidime (CAZ), ciprofloxacin (CIP), trimethoprim-sulfamethoxazole (SXT), cefoperazone/sulbactam (SFP), cefepime (FEP), tobramycin (TOB), piperacillin-tazobactam (TZP), imipenem (IPM), meropenem (MEM), minocycline (MIN), tigecycline (TGC), amikacin (AMK), doxycycline (DOX), levofloxacin (LVX), ampicillin/sulbactam (SAM), and colistin (CST) were determined using the broth microdilution method according to the guidelines of the Clinical and Laboratory Standards Institute (CLSI, 2020). The Food and Drug Administration (FDA)^1^ breakpoints were used if CLSI breakpoints were not available.

### 2.3 Whole-genome sequencing and data analysis

Genomic DNA from each isolate from the pregnant women was extracted using the PureLink genomic DNA mini kit (Invitrogen, United States). The bacterial genomes were sequenced using the Illumina HiSeq 2500 platform (Illumina, San Diego, CA, United States), and fastp was used to remove low-quality, low-complexity reads and polyG/polyX tails (Chen et al., 2018). The genomes were assembled with *de novo* SPAdes Genome Assembler (version 3.15.2; Antipov et al., 2016), and the assembled genomes were annotated using Prokka (1.13.2; Seemann, 2014). Resistance genes and virulence factors were identified using ResFinder 4.1^2^ and VFDB^3^ databases, respectively.

The types of KL and OCL synthesis were identified using Kaptive software (Wyres et al., 2020). MLST was performed based on both Oxford and Pasteur schemes (Bartual et al., 2005; Diancourt et al., 2010), and genome sequences were compared with nucleotide sequences of housekeeping genes in the MLST database^4^ to determine the number of alleles and assign sequence types (STs). Clonal complexes (CCs) were defined as groups of STs isolates sharing at least 6/7 alleles. The cgMLST scheme consisting of 2,390 conserved genome-wide genes was performed with Ridom SeqSphere+ (version 5.1.0; Higgins et al., 2017). Genomes containing at least 90% of the defined cgMLST targets were included, and closely related genomes with fewer than 10 different alleles in the cgMLST target gene set were considered highly related as a unique nomenclature of complex type (CT) or cluster (Higgins et al., 2017). Core genome SNP (cgSNP) analysis was performed using Parsnp (version 1.5.4) from the Harvest suite (Treangen et al., 2014) with default parameters, with the exception of parameter-c. The strain *A. baumannii* S1 (SAMN10618186) was selected as the reference genome and the total number of SNPs was obtained using HarvestTools. The online tool iTOL was used to display, manipulate, and annotate phylogenetic trees.^5^ The genome sequences were deposited in GenBank under BioProject PRJNA779035.

### 2.4 *Galleria mellonella* larva infection assay

The virulence potential of AB was examined in the infection model of *G. mellonella* larvae (Tianjin Huiyude Biotech Company, Tianjin, China), as previously described (Peleg et al., 2009). Briefly, overnight cultures of AB single colony were adjusted to 1 × 10^8^ colony-forming unit (CFU)/ml with phosphate-buffered saline (BPS). After injection with 0.01 mL of the above bacterial suspension, the larvae were incubated in the dark at 37°C. The survival rate of the *G. mellonella* was recorded for 5 days. All experiments were technically repeated three times with triplicate biological replication (*n* = 60 larvae per condition). BPS and *A. baumannii* ATCC19606 were included as control groups.

### 2.5 Statistical analysis

Univariate and multivariate logistic regression analyses were performed to identify potential influencing factors from pregnant women associated with CRAB carriage. Odds ratios (OR) and 95% confidence intervals (CI) were examined. A *p* value of <0.05 was considered statistically significant. The survival curve was constructed using the Kaplan–Meier method, with *p* values calculated using the log-rank test. Actual agreement between groups of different schemes was assessed using Cohen’s weighted kappa (κ_w_) coefficients. Pearson’s chi-square exact test (two-tailed) was used to compare the differences. Statistical analyses were performed with SPSS (version 28.0).

## 3 Results

### 3.1 Characteristics of the study participants

In total, 138 pregnant women participated in the ASC program, with 36 (26.09%) testing positive for carrying the CRAB. The data revealed no carbapenemase-producing *Pseudomonas aeruginosa* carriers, three carbapenemase-producing *Escherichia coli* carriers, and 17 (12.32%) carbapenem-resistant *Klebsiella pneumoniae* (CRKP) carriers. Logistic regression analyses were performed on 136 participants with available clinical data; Table 1 illustrates a comparative analysis of clinical characteristics between carriers and non-carriers.

**Table 1:** Univariate and multivariate analysis of risk factors for carbapenem-resistant *Acinetobacter baumannii* carriage in pregnant women.

Characteristic*^a^*	Total no. of pregnant women*^b^*	No. of CRAB carriers*^c^*	% of CRAB carriers	Univariate analysis*^d^*			Multivariable analysis*^d^*		
				OR	95% CI	*p* value	OR	95% CI	*p* value
*Advanced maternal age*									
Yes (≥ 35 years)	25	11	44.00	2.70	1.09–6.69	0.032	3.63	1.27–10.33	0.016
No	111	25	22.52						
*Registered residence*									
Guangdong	70	21	30.00	1.46	0.67–3.15	0.338			
Outside Guangdong	66	15	22.73						
*No. of pregnancy*									
Primigravida	54	15	27.78	1.10	0.51–2.39	0.812			
Multigravida	81	21	25.93						
*History of vaginal delivery*									
Yes	40	8	20.00	0.59	0.24–1.44	0.245			
No	94	28	29.79						
*Cesarean section history*									
Yes	29	11	37.93	1.96	0.82–4.69	0.133			
No	105	25	23.81						
*History of miscarriage/abortion*									
Yes	40	11	27.50	1.03	0.45–2.37	0.941			
No	93	25	26.88						
*Pre-pregnancy BMI (kg/m^2^)*									
Normal weight (18.5–23.99)	85	24	28.24						
Underweight (< 18.5)	21	4	19.05	0.60	0.18–1.96	0.396			
Overweight (24 ≤)	25	7	28.00	0.99	0.37–2.67	0.982			
*No. of ANC visits*									
Moderate (8–10)	35	9	25.71						
Low (≤ 7)	31	7	22.58	0.84	0.27–2.62	0.767			
High (≥ 11)	67	20	29.85	1.23	0.49–3.09	0.660			
*Anemia in pregnancy*									
Yes (hemoglobin <11 g/dL)	54	11	20.37	0.61	0.27–1.37	0.231			
No	81	24	29.63						
*Diabetes mellitus*									
Yes (pre-pregnancy diabetes)	5	2	40.00	2.89	0.45–18.58	0.264	2.58	0.35–19.17	0.355
Yes (GDM)	35	16	45.71	3.65	1.58–8.45	0.003	4.88	1.89–12.60	0.001
No	96	18	18.75						
*Thyroid disease*									
Yes	21	9	42.86	2.45	0.93–6.42	0.070			
No	115	27	23.48						
*Hypokalemia*									
Yes	12	4	33.33	1.44	0.41–5.1	0.574			
No	124	32	25.81						
*Hypocalcemia*									
Yes	9	5	55.56	3.87	0.98–15.32	0.054			
No	127	31	24.41						
*HBV status*									
HBV positive	11	6	54.55	3.8	1.08–13.34	0.037	2.51	0.60–10.55	0.207
HBV negative	125	30	24.00						
*HSV status*									
HSV positive	8	4	50.00	3.00	0.71–12.69	0.136			
HSV negative	128	32	25.00						
*GBS infection*									
Reproductive tract positive	10	7	70.00	7.80	1.9–32.12	0.004	12.75	2.72–59.87	0.001
Reproductive tract negative	126	29	23.02						
*Mycoplasma*									
Reproductive tract positive	6	3	50.00	2.94	0.57–15.28	0.200			
Reproductive tract negative	130	33	25.38						
*Oropharyngeal swab test*									
CRKP positive	17	6	35.29	1.62	0.55–4.75	0.381			
CRKP negative	119	30	25.21						

^*a*^BMI, body mass index; ANC, antenatal care; GDM, gestational diabetes mellitus; HBV, hepatitis B virus; HSV, herpes simplex virus; GBS, Group B *Streptococcus*; CRKP, carbapenem-resistant *Klebsiella pneumoniae*.

^*b*^The number of pregnancies for one pregnant woman was unknown; two pregnant women had no history of vaginal delivery or cesarean section; three pregnant women had no history of miscarriage/abortion; five pregnant women had no pre-pregnancy BMI information; three pregnant women had no data on the number of ANC visits; the hemoglobin value of one pregnant woman was unknown on the date of hospital admission.

^*c*^The number of ANC visits of one pregnant woman with carriage of CRAB was unknown; the hemoglobin value of one pregnant woman with carriage of CRAB was unknown.

^*d*^OR, odds ratio; 95% CI, 95% confidence interval.

Univariate analysis identified risk factors for the oropharyngeal carriage of CRAB at admission, including advanced maternal age (AMA; OR, 2.70; *p* = 0.032), gestational diabetes mellitus (GDM; OR, 3.65; *p* = 0.003), being Hepatitis B Virus (HBV)-positive (OR, 3.8; *p* = 0.037), and testing positive for group B *Streptococcus* (GBS) in the reproductive tract (OR, 7.80; *p* = 0.001). Moreover, the multivariate analysis highlighted advanced maternal age (OR, 3.63; *p* = 0.016), GDM (OR, 4.88; *p* = 0.001), and GBS positivity (OR, 12.75; *p* = 0.001) as statistically significant associative factors for carriage. After the initial identification, five carriers were rehospitalized and resampled due to recurrent or alternative clinical symptoms, as detailed in Table 2. The reason for hospitalization for each pregnant woman is presented in the Supplementary Table 2. Notably, no participants or their newborns succumbed to a CRAB infection, according to the related information from medical records and participant follow-up processes.

**Table 2:** Pregnant women and related isolate characteristics at two-time points.

Case	Gestational age*^a^*	Admitting diagnosis*^b^*	Maternal characteristics*^c^*	Sample ID	CT*^d^*	Resistance phenotype*^e^*	AR genes*^f^*	Virulence genes*^g^*
1	8 + 1	Light vaginal bleeding for 9 days	**Scarred uterus, CRKP carrier**, SCH	PWa10	3071	**SXT^r^, SFP^r^, FEP^r^, TOB^r^, TGC^s^**, MIN^s^	***armA, bla_TEM-1D_, mph(E), msr(E), sul2***	***csuE, abaR***
	39 + 2	Oligohydramnios on admission day	**Scarred uterus, CRKP carrier**, Pelvic adhesion	PWa41	3071	**SXT^r^, SFP^r^, FEP^r^, TOB^r^, TGC^s^**, MIN^i^	***armA, bla_TEM-1D_, mph(E), msr(E), sul2***	***csuE, abaR***
2	28 + 4	Elevated bile acids for 2+ months, vaginal bleeding on admission day	**Scarred uterus, GDM, Hepatitis B, Hyper-bileacidaemia, CRKP carrier**	PWb42	512	**SFP^r^, TOB^r^, MIN^r^, TGC^s^**, SXT^s^, FEP^r^	***armA, mph(E), msr(E)***	***csuE***
	35 + 2	Elevated bile acids for 3+ months	**Scarred uterus, GDM, Hepatitis B, Hyper-bileacidaemia, CRKP carrier**, Pelvic adhesion	PWa26	2092	**SFP^r^, TOB^r^, MIN^r^, TGC^s^**, SXT^r^, FEP^i^	***armA, mph(E), msr(E)***	***csuE***
3	29 + 6	High S/D ratio by Doppler US on admission day	**Scarred uterus, HSV infection, Immune disorders, Chronic pharyngitis**, Vitamin D deficiency	PWa43	3136	**SXT^r^, SFP^r^, FEP^r^, TOB^r^, MIN^r^, TGC^r^**	***armA, bla_TEM-1D_, mph(E), msr(E)****, sul1, aac(6*′*)-Ib3, aac(6*′*)-Ib-cr, aadA1, catB8, qacE*	***csuE, abaR***
	33 + 2	Abnormal umbilical artery blood flow 1+ day, abnormal result of fHR monitoring on admission day	**Scarred uterus, HSV infection, Immune disorders, Chronic pharyngitis**, Chorioamnionitis, CRKP carrier	PWb15	3071	**SXT^r^, SFP^r^, FEP^r^, TOB^r^, MIN^r^, TGC^r^**	***armA, bla_TEM-1D_, mph(E), msr(E)***, *sul2*	***csuE, abaR***
4	37 + 6	Abnormal result of fetal heart rate monitoring on admission day	**GDM**	PWa57	3071	**SXT^r^, SFP^r^, FEP^r^, MIN^i^**, TOB^r^, TGC^s^	***bla_TEM-1D_, sul2*** *, armA, mph(E), msr(E)*	*csuE, abaR*
	39 + 2	Vaginal bleeding with lower abdominal pain for 3+ hours	**GDM**	PWb10	2092	**SXT^r^, SFP^r^, FEP^r^, MIN^i^**, TOB^s^, TGC^r^	***bla_TEM-1D_, sul2***	
5	31 + 2	Painless vaginal bleeding for 1+ hour	**Uterine fibroids, Velamentous placenta, Hepatitis B, AMA**	PWa72	3071	**FEP^r^, TOB^r^**, SXT^s^, SFP^i^, MIN^i^, TGC^r^	***armA, mph(E), msr(E)***, *bla_TEM-1D_, sul2*	***csuE*** *, abaR*
	36 + 1	Painless vaginal bleeding for 1+ hour	**Uterine fibroids, Velamentous placenta, Hepatitis B, AMA**, Anemia in pregnancy	PWb5	512	**FEP^r^, TOB^r^**, SXT^s^, SFP^i^, MIN^i^, TGC^r^	***armA, mph(E), msr(E)***	***csuE***

^*a*^Weeks + day(s).

^*b*^S/D, peak systolic blood flow velocity/end-diastolic blood flow velocity; US, ultrasound; fHR, fetal heart rate.

^*c*^CRKP, carbapenem-resistant *Klebsiella pneumoniae*; SCH, subchorionic hematoma; GDM, gestational diabetes mellitus; HSV, herpes simplex virus; AMA, advanced maternal age; maternal characteristics which are the same at both two-time points during a gestation period of one host is shown in bold.

^*d*^CT, the complex type determined using cgMLST by Ridom SeqSphere+.

^*e*^The 10 related isolates were all resistant to ceftazidime (CAZ), ciprofloxacin (CIP), piperacillin-tazobactam (TZP), imipenem (IPM), meropenem (MEM), amikacin (AMK), doxycycline (DOX), levofloxacin (LVX), ampicillin/sulbactam (SAM); susceptible to colistin (CST); SXT^r^, resistant to trimethoprim-sulfamethoxazole; SXT^s^, susceptible to SXT; SFP^r^, resistant to cefoperazone/sulbactam; SFP^i^, intermediate to SFP; SFP^s^, susceptible to SFP; FEP^r^, resistant to cefepime; FEP^i^, intermediate to FEP; FEP^s^, susceptible to FEP; TOB^r^, resistant to tobramycin; TOB^s^, susceptible to TOB; MIN^r^, resistant to minocycline; MIN^i^, intermediate to MIN; MIN^s^, susceptible to MIN; TGC^r^, resistant to tigecycline; TGC^s^, susceptible to TGC.

^*f*^AR, antibiotic resistance. The 10 related isolates all carrying *aph(3*′*)-Ia, aph(3*″*)-Ib, aph(6)-Id, bla_ADC-25_, bla_OXA-23_, bla_OXA-66_, tet(B)*. The gene in bold is carried by both isolates from the same pregnant woman at two-time points.

^*g*^The 10 related isolates all carrying *ompA, adeF, adeG, adeH, bap, csuA/B, csuA, csuB, csuC, csuD, pgaA, pgaB, pgaC, pgaD, plc, plcD, lpsB, lpxA, lpxB, lpxC, lpxD, lpxL, lpxM, barA, barB, basA, basB, basC, basD, basF, basG, basH, basI, basJ, bauA, bauB, bauC, bauD, bauE, bauF, entE, hemO, abaI, bfmR, bfmS, pbpG, katA*; The gene in bold is carried by both isolates from the same pregnant woman at two-time points.

### 3.2 Characteristics of oropharyngeal isolates in pregnancy

From 143 oropharyngeal samples obtained from the 138 participants, 41 CRAB isolates were identified. These isolates demonstrated resistance to a majority of common antibiotics but showed susceptibility to colistin. A notable percentage was intermediate to minocycline (*n* = 21, 51.22%) and susceptible to tigecycline (*n* = 27, 65.85%; Supplementary Table 3). Based on cgMLST, 13 CTs, including four newly created CTs, i.e., CT3193 (2.44%, 1/41), CT3071 (36.59%, 15/41), CT3136 (7.32%, 3/41), and CT3135 (2.44%, 1/41), were distributed in 4 Clusters^RS^ and one orphan from isolates during pregnancy (Table 3). The predominant group was Cluster^RS^ 1, followed by Cluster^RS^ 13, among the 4 Clusters^RS^. Detailed distribution of resistance- and virulence-related genes, genotypes, and the geographic distribution of Clusters^RS^ are elaborated in Supplementary Table 3 and Supplementary Figure 1. Although Cluster^RS^ 13 was a relatively minor cluster in the worldwide dataset (*n* = 77), all of its isolates shared the same type of KL49 with the hypervirulence strain LAC-4. The pathogenicity of selected different KL-type isolates was evaluated by performing *in vivo* virulence experiments in *G. mellonella* injection larvae. The survival rates of the KL49 isolates were significantly lower than those of the non-KL49 isolates (Supplementary Figure 2; *p* < 0.001 by log-rank test).

**Table 3:** Genotypic distribution of carbapenem-resistant *A. baumannii* carriage among pregnant women in the present study.

cgMLST*^a^*		Kaptive*^b^*		MLST*^c^*				% in study vs. database*^d^*							
Cluster^RS^ (no. of isolates, % in this study)	CT (no. of isolates, % in the cluster of study)	KL	OCL	ST^OX^	CC^OX^	ST^IP^	CC^IP^	Cluster	CT	KL	OCL	ST^OX^	CC^OX^	ST^IP^	CC^IP^
Cluster 1 (*n* = 31, 75.61)	715 (*n* = 2, 6.45)	KL3	OCL1	195/1816	208	2	2	3.18	1.61	0.51	0.66	0.87	0.72	0.75	0.72
	1044 (*n* = 1, 3.23)	KL3	OCL1	195/1816	208	2	2		3.03						
	2085 (*n* = 1, 3.23)	KL3	OCL1	195/1816	208	2	2		7.69						
	2087 (*n* = 3, 9.68)	KL3	OCL1	195/1816	208	2	2		50						
	2907 (*n* = 1, 3.23)	KL3	OCL1	195/1816	208	2	2		33.33						
	3139 (*n* = 1, 3.23)	KL3	OCL1	195/1816	208	2	2		100						
	2082 (*n* = 7, 22.58)	KL77	OCL1	136/1851	208	2	2		24.14	4.61		8.05			
	3071 (*n* = 15, 48.39)	KL7	OCL1	208/1806	208	2	2		100	4.36		1.07			
Cluster 40 (*n* = 3, 7.32)	3136 (*n* = 3, 100)	KL2	OCL1	208/1806	208	2	2	13.04	100	0.31					
Cluster 225 (*n* = 1, 2.44)	2088 (*n* = 1, 100)	KL2	OCL1	208/1806	208	2	2	20	20						
Orphan (*n* = 1, 2.44)	3135 (*n* = 1, 100)	KL2	OCL1	1905/New86	208	2	2		100			50			
Cluster 13 (*n* = 5, 12.20)	512 (*n* = 3, 60)	KL49	OCL1	457/1891	208	2	2	6.49	4.55	1.59		5.95			
	2092 (*n* = 2, 40)	KL49	OCL1	457/1891	208	2	2		25						

^*a*^cgMLST, core genome multilocus sequence typing; Cluster^RS^, cluster determined using cgMLST by Ridom SeqSphere+.

^*b*^Kaptive is a tool for bacterial surface polysaccharide locus typing and variant evaluation; KL, capsular polysaccharide; OCL, lipooligosaccharide outer core.

^*c*^MLST, multilocus sequence typing; ST^OX^, sequence type determined using the Oxford scheme; New86, one of the new STs of the Oxford scheme; CC^OX^, clonal complex from the Oxford scheme; ST^IP^, ST of the Pasteur scheme; CC^IP^, CC from the Pasteur scheme.

^*d*^The database used in this study contains 9,470 genomes in total with available isolate-related information, except for 41 from pregnant women in the present study, 9,429 assembled from NCBI Sequence Read Archive (SRA) and downloaded from NCBI Reference Sequences (RefSeq).

### 3.3 Evolutionary history and phylogenetic relationships of KL49 *Acinetobacter baumannii*

**Figure 1:**
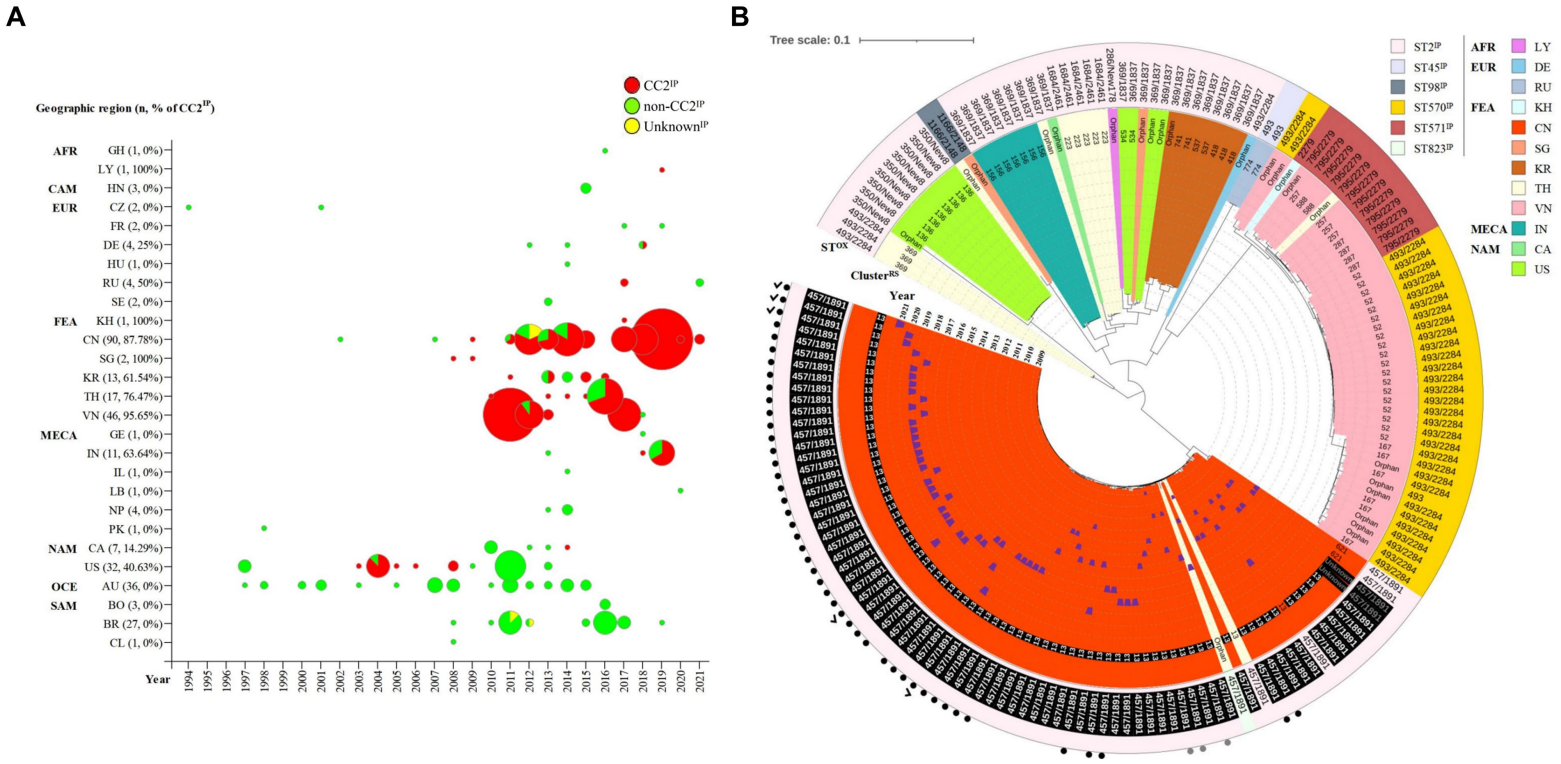
Spatiotemporal distribution and phylogeny of KL49 *Acinetobacter baumannii* isolates. **(A)** In total, 314 KL49 *A. baumannii* isolates were collected in the database, divided into CC2, non-CC2, and Unknown groups using the Pasteur MLST scheme, and they were distributed in Africa (AFR), including Ghana (GH) and Libya (LY); Central America (CAM), including Honduras (HN); Europe (EUR), including the Czech Republic (CZ), France (FR), Germany (DE), Hungary (HU), Russia (RU), and Sweden (SE); Far-East Asia (FEA), including Cambodia (KH), China (CN), Singapore (SG), South Korea (KR), Thailand (TH), and Vietnam (VN); the Middle East and Central Asia (MECA), including Georgia (GE), India (IN), Israel (IL), Lebanon (LB), Nepal (NP), and Pakistan (PK); North America (NAM), including Canada (CA) and the USA (US); Oceania (OCE), including Australia (AU); and South America (SAM), including Bolivia (BO), Brazil (BR), and Chile (CL). In terms of time axis, the worldwide spread of KL49 *A. baumannii* isolates occurred over the 28 years from 1994 to 2021 in the present study. **(B)** The phylogenetic tree constructed on the cgMLST of 172 CC^IP^2-KL49 *A. baumannii* isolates from 12 countries was performed with Ridom SeqSphere+ and visualized using iTOL. Tree branches were color-coded to highlight different countries. The number or word “orphan” at the end of each branch showed that the cluster of isolation or orphan was defined by cgMLST using Ridom SeqSphere+ (version 5.1.0). The numbers of Cluster^RS^ 13 and ST457/1891^OX^ written in white font and shaded as black boxes represent isolates from Guangdong province, China. The words “Unknown” and ST457/1891^OX^ are written in gray font and shaded as black boxes, representing isolates from a region with unknown geographic information in China. The year of collection of the corresponding isolate was added and is marked with blue trapezoids in the sector of the inner circle with the color that represents CN. Different background colors and different Pasteur STs^IP^ correspond to each other in the outermost circle. The numbers depict the corresponding Oxford STs^OX^ in the outermost circle. The solid black dots and solid gray dots outside the outermost circle represent isolates from Shenzhen and regions with unknown geographic information in Guangdong, China. The check marks outside the outermost circle represent isolates from this study.

The global distribution of 314 KL49 isolates is presented in Supplementary Figure 3 and Figure 1A, with 54.78% (172/314), 43.95% (138/314), and 1.27% (4/314) being assigned to CC2^IP^, non-CC2^IP^, and Unknown^IP^, respectively, using the Pasteur MLST scheme. This scheme demonstrated high concordance with Bayesian population structure analyses on 2,390-loci cgMLST (Supplementary Figure 3). Though the KL49 isolate was initially reported in 1994 in the Czech Republic and subsequently identified in various global regions, CC2^IP^-KL49 isolates have been predominantly found in Far-East Asia, accounting for 85.47% (147/172) of identifications. Specifically, CC2^IP^-KL49 isolates were first found in pregnant women and evolved to be hypervirulent with multiple antibiotics resistance genes.

**Figure 2:**
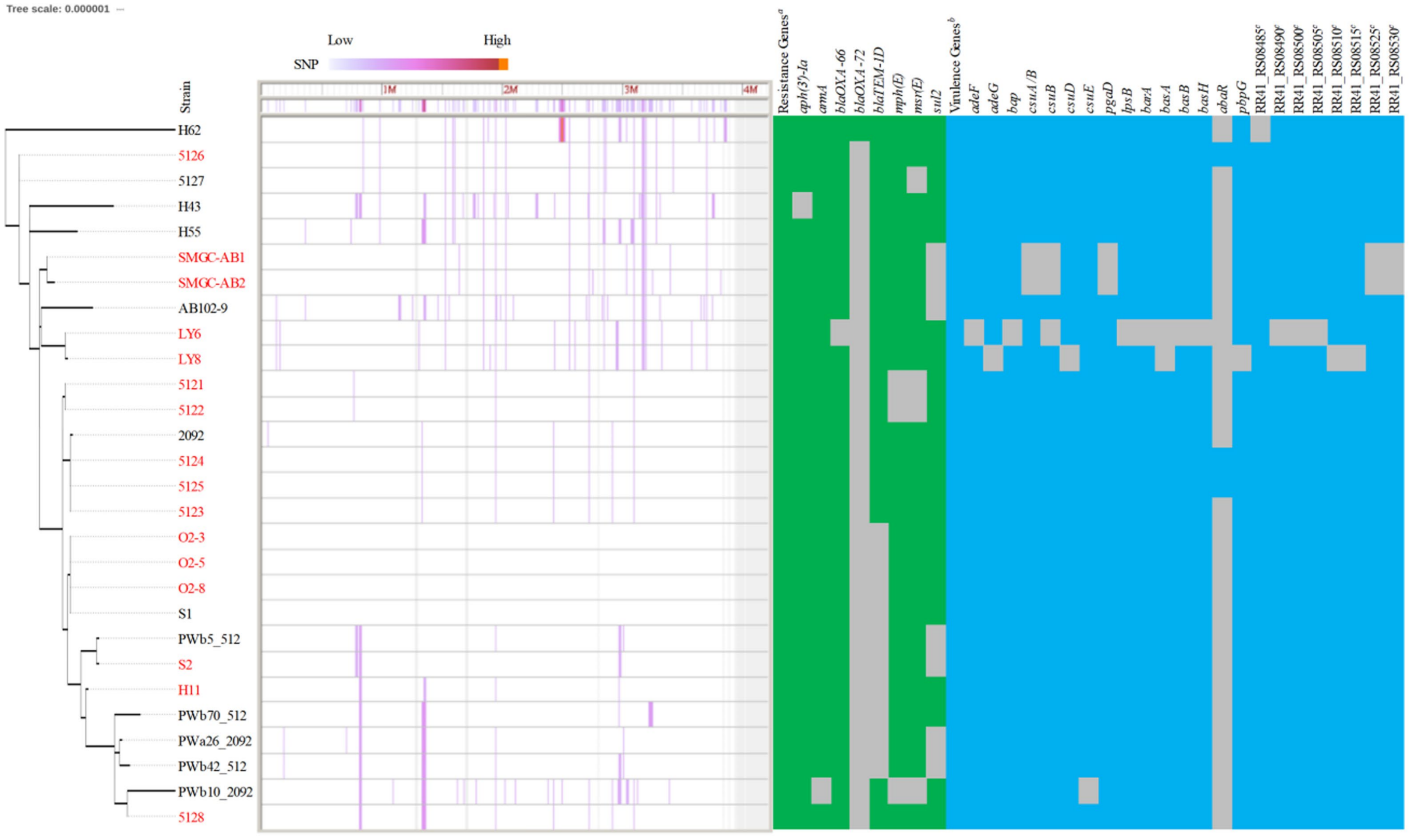
Characteristics of 28 CC2^IP^-KL49 *Acinetobacter baumanii* isolates with the information of patient outcome. Strain names in red indicate that the corresponding patient died; strain names in black indicate that the corresponding patient survived. Phylogenomic analysis was based on core genome SNP using Parsnp. The degree of color represents the SNP density. The genome of strain S1 was used as a reference. Identification of the resistance genes was carried out with sequences from the ResFinder database (e-value cutoff 1 × E^−5^, coverage ≥0.9, identity ≥0.9). Positive matches are indicated with green rectangles; negative matches are shown as gray spaces. The virulence genes were identified using the VFDB database (e-value cutoff 1 × E^−5^, coverage ≥0.9, identity ≥0.9), with positive matches indicated by blue rectangles and negative matches shown as gray spaces. *^a^*The 28 isolates all carrying the resistance genes, including *aph(3″)-Ib*, *aph(6)-Id*, *bla_ADC-25_*, *bla_OXA-23_*, *tet(B)*. ^*b*^The related isolates all carrying the virulence genes, including *ompA, adeH, csuA, csuC, pgaA, pgaB, pgaC, plc, plcD, lpxA, lpxB, lpxC, lpxD, lpxL, lpxM, barB, basC, basD, basF, basG, basI, basJ, bauA, bauB, bauC, bauD, bauE, bauF, entE, hemO, abaI, bfmR, bfmS, katA*, and RR41_RS08495 (a gene involved in the heme utilization of strain LAC-4). *^c^*A set of genes involved in the heme utilization of strain LAC-4.

Compared with MLST’s ability to type 172 CC2^IP^-KL49 AB, cgMLST separated isolates into several distinct clades (Figure 1B). The major clade consisting of 81 isolates that were assigned to Cluster^RS^ 13 (*n* = 76), Cluster^RS^ 621 (*n* = 2), Unknown^RS^ (*n* = 2), and Orphan^RS^ (*n* = 1), corresponding to 100% ST457/1891^OX^ isolates, was mainly distributed in China (97.53%, 79/81). Guangdong province represented 91.36% of the 81 total isolates. The phylogeographical specificity of each Cluster^RS^ in the KL49 database isolates is illustrated in Figure 1B and Supplementary Figure 3. Their deeper genetic differences among 28 CC2^IP^-KL49 isolates, with the information on patient outcome, were further investigated through cgSNP analysis, with a presence/absence table based on the resistance- and virulence-related genes, for visualization alongside the phylogenetic tree (Figure 2). There was no obvious clade consisting of the different groups with CC2^IP^-KL49 isolates. In particular, the T6SS with various deletions was identified in the CC2^IP^-KL49 isolates. In contrast, all the core genes of the T6SS were identified in the KL7 isolates, i.e., CT3071 isolates, which were also common in the five participants with dual isolates.

## 4 Discussion

A database selection of 114 isolates from Shenzhen in Guangdong Province, excluding the 41 isolates from this study, showcased that 59 (51.75%, 59/114) isolates belonged to Cluster^RS^ 1, followed by Cluster^RS^ 13 (35.09%, 40/114) and Cluster^RS^ 225 (3.51%, 4/114). Intriguingly, 90.35% (*n* = 103) of the total isolates shared the same Clusters^RS^ with the isolates found in pregnant women from Shenzhen, indicating that the genotypes of carriage isolates during pregnancy were the local prevalent AB or their closely related pathogens. However, a much higher proportion of Cluster^RS^ 1 isolates was observed among pregnant women compared with the previous proportion (75.61% vs. 51.75%; χ^2^, 7.05; *p* < 0.01) in Shenzhen. Independent of whether cgMLST was performed exclusively on the 41 isolates in the current study or all 155 isolates in Shenzhen, the 31 Cluster^RS^ 1 isolates from pregnant women could be subdivided into three minor clusters, with CT3071 and CT2082 isolates forming an independent cluster (Supplementary Figure 4). Although there were at least 15 different alleles between the CT3071 isolates and other Cluster^RS^ 1 isolates in Shenzhen, an isolate with only five different alleles was identified in Guangzhou, a city adjacent to Shenzhen in Guangdong province. Cluster^RS^ 1, the largest Cluster^RS^ and host to substantial genetic diversity globally, had over half its isolates with novel CTs in this study. Upon excluding novel CTs isolates present only during pregnancy, the CRAB oropharyngeal carriage rate in pregnant women was halved to 13.04%, which was a rate similar to previous studies in the United States (13.46%, 49/364; Latibeaudiere et al., 2015), South Korea (15.06%, 168/1115; An et al., 2017), and Japan (13.52%, 172/1272; Yamamoto et al., 2019).

Several risk factors enhance the likelihood of CRAB carriers during pregnancy, aligning with findings from the general population (Alsultan et al., 2013; Ren et al., 2019; Diep et al., 2023). AMA has previously been shown to increase the risk of adverse pregnancy outcomes, maternal death, and maternal complications, such as GDM (Alsultan et al., 2013; Laopaiboon et al., 2014; Fink et al., 2023), and was suggested as an independent risk factor for CRAB carriers. Diabetes mellitus has been demonstrated as a significant risk factor for AB infections, and diabetic patients are significantly more likely to carry CRAB (Alsultan et al., 2013; Ren et al., 2019). It has previously been revealed that diabetes could destroy the control relationships between bacteria, disrupting the human micro-ecological balance, which is conducive to forming pan-resistant species of AB (Ren et al., 2019). The number of pregnant women with pre-existing diabetes was relatively low in the present study, whereas GDM was found to be an independent risk factor for pregnant women who carried the CRAB isolates; GDM also resulted in an increased risk of complications, such as leading to the development of type 2 diabetes in both the mother and infant (Guevara-Ramírez et al., 2023). Infections in pregnancy are common and are associated with numerous consequences for the mother, fetus, and neonate (Hazenberg et al., 2021). GBS infection, which was identified by VITEK 2, VITEK MS, and post-enrichment subculture methods in this study, also stood out as a notable independent risk factor for the carriage of CRAB in pregnant women. There was no high-risk group associated with certain Cluster^RS^ or CT isolates.

It is widely known that pathogen carriage is strongly associated with the subsequent development of infections. Although the results in the present study were consistent with previously published observations that there were no adverse maternal and neonatal effects due to AB during pregnancy (Sood et al., 2019), the presence of CR-hvAB with KL49 isolates could indicate that special internal environments or evolutionary strains control human micro-ecological balance and counterbalance this high pathogenicity and mortality during pregnancy. Hormonal alterations and reduced immunity during pregnancy confer a greater risk of emerging or resurgent infectious diseases. Meanwhile, there are many measures to help pregnant women fight disease, such as the supplementation of omega-3 polyunsaturated fatty acids during pregnancy, which has exhibited superior antimicrobial effects against the highly drug-resistant pathogen AB (Jiang et al., 2019).

Shenzhen is witnessing a surge in migrant population and is grappling with ensuing public health challenges such as healthcare service inequity and escalating infectious diseases, especially among vulnerable demographics such as pregnant women (Wang et al., 2023). The first KL49 AB (BioProject PRJNA388510) was found in the Third People’s Hospital of Shenzhen in 2014. Notably, a mother who had just given birth to a newborn baby played a central role in a fatal outbreak of KL49 strains at Shenzhen People’s Hospital in the Luohu district, with a deadly 77.78% mortality rate in 2017 (BioProject PRJNA533558; Supplementary Figure 5). The following year, KL49 strains were implicated in another severe outbreak with an 80% mortality rate, involving two departments, and the transmission was associated with contamination of the bedside chest roentgenogram machine (BioProject PRJNA510897); the KL49 strain was also identified in the Intensive Care Unit (ICU) portable ventilator (BioProject PRJNA546440). Of the 286 AB clinical isolates from the Department of Clinical Laboratory identified using a PCR rapid detection method (Deng et al., 2020), 53 (18.53%) KL49 isolates were detected across 11 departments between 2017 and 2019 at Shenzhen People’s Hospital. A total of 489 patients were incorporated on admission (<2 h) in the ICU at Shenzhen People’s Hospital for an ASC program, which revealed that 177 and 156 carbapenemase-producing organisms *Acinetobacter* isolates were oropharyngeal and intestinal carriage, respectively, from January to November 2019. To date, there has been less effort to monitor AB carriage in pregnancy.

KL49 AB isolates are associated with fatal outbreaks or severe infections in relatively immunocompetent patients; their high virulence was not only observed in clinical data, but also reproduced in animal models, including lethal to immunocompetent mice and *G. mellonella* (Jones et al., 2015; Zhou et al., 2018; Zeng et al., 2019; Deng et al., 2020; Li et al., 2020). The CC2^IP^-KL49 isolates were divided into 16 Clusters^RS^; among them, Cluster^RS^ 13 was the predominant group, accounting for 44.19% (76/172). In contrast to Cluster^RS^ 1, as “generalist pathogens”, able to adapt to several human populations and spread around the world, Cluster^RS^ 13 demonstrated surprising phylogeographical specificity to Guangdong province (Supplementary Figures 1, 3; Figure 1A). This geographical restriction could correspond to the local adaptation of these “ecological specialist” KL49 isolates to local human host populations. Although both previous studies in Guangdong, China, and many parts of the world outside of Guangdong have demonstrated that Cluster^RS^ 13 and other Cluster^RS^ KL49 isolates are more virulent than non-KL49 (Jones et al., 2015; Zhou et al., 2018; Deng et al., 2020; Li et al., 2020), the Cluster^RS^ 13 isolates did not support its high virulence to cause significant morbidity and mortality in pregnant women. In addition to host factors, no specific evolution of KL49 strains has been identified in pregnant women through multiple genotyping and the differential analysis of SNPs comparing KL49 strains with hosts’ death and survival information.

Two extensively drug-resistant AB, with significantly different mortality rates in the *G. mellonella* infection model, obtained at two-time points during a case, have previously been reported (Grier et al., 2021). In the present study, there were five participants from whom two samples were obtained at different interval times. The five pairs of isolates displayed more or less distinct genomic or phenotypic characteristics in comparison to two strains within the same host, including some critical drug-resistance phenotypes, which was also reported in a previous study of two AB isolates from a single patient, with subsequent change from tigecycline susceptibility to resistance (Hornsey et al., 2011). In general, the co-occurrence of two highly resistant bacteria in the same patient may lead to therapeutic failure and may lead to death (Ding et al., 2021). However, dual infections of two clinical strains of CRAB have been identified in the same sample from a patient (Hadjadj et al., 2018). In the present study, KL49 isolates and KL7 isolates (i.e., CT3071 isolates in pregnancy) were most common in the five participants with two isolates, accounting for 50% and 40% of the 10 total related CRAB isolates, respectively (Table 2).

The population bias for CT3071 was similar to a prior report that K14.K64-CRKP mainly occurred in adult patients, especially patients >65 years of age, and wzi209-CRKP mainly occurred in pediatric patients, especially infants (Zhu et al., 2022). The CT3071 isolates exhibiting KL7, as the most prevalent CRAB isolates of maternal carriage, were potentially able to adapt to the population of pregnant women (Table 3). The capsule thickness of the KL7 isolates was significantly higher than that of other capsular types (Hu et al., 2020), which was identified in Shenzhen for the first time. Compared with KL49 isolates, CT3071 isolates possess a T6SS that has the ability to mediate the killing of bacterial competitors (Jones et al., 2015; Zhou et al., 2018; Zhang et al., 2023). Considering CT3071 isolates have lower virulence than KL49 isolates (Supplementary Figure 2), their potential intraspecific competition might also be a possible reason for the absence of morbidity and mortality in pregnant women.

The limitations of this study prominently include the singular sampling site and hospital from which isolates were collected, especially on account of the multiple sampling populations and sites are important to compare and confirm the results, and the disruption caused by the COVID-19 pandemic affecting screening and participant follow-up processes. Such clinical information as, history of previous hospitalization (pregnancy-unrelated), smoking, and inappropriate nutritional status, were unavailable for analysis, since there was no positive carrier among the related populations in this small sample size. It is also likely that because of the strict disinfection procedure and widespread mask-wearing habits during the COVID-19 pandemic in China, no related genotype of the AB strain in pregnancy was found in the environment or healthcare workers during this study. Therefore, there was no direct evidence to indicate whether the origins of the relevant strains in pregnant women were from the hospital, the community environment, or other transmission chains, particularly the newly created CTs isolates. Despite these limitations, the findings in the present study are significant since, in the potential chain of mother-to-newborn transmission, all are very susceptible to infections, with quite limited antibiotics for therapeutic options, particularly in the face of previous fatal neonatal outbreaks and contamination over animate and inanimate environment (Osei Sekyere et al., 2021; Odoyo et al., 2023; Woon et al., 2023). In China and most developing countries, there needs to be more individual rooms to take contact precautions and enforce patient isolation in the Department of Obstetrics and Gynecology, especially in public hospitals, which is an issue that deserves more attention.

## 5 Conclusion

This is the first study to describe the situations of the carriage of CRAB among pregnant women in China. The independent risk factors for carriers included AMA, GDM, and GBS infection during pregnancy. The discovery of a significant proportion of newly created CT isolates during pregnancy is noteworthy and demands further study to decode pathogen-pathogen compatibility, understand the characteristics of emerging genotypes such as CT3071, and develop a broader surveillance network of CRAB isolates during pregnancy for effective detection and control in both hospital and community settings, especially in Guangdong for the hypervirulent KL49 isolates.

## Data availability statement

The datasets presented in this study can be found in online repositories. The names of the repository/repositories and accession number(s) can be found in the article/Supplementary material.

## Ethics statement

The studies involving humans were approved by the Medical Ethics Committee of Shenzhen People’s Hospital. The studies were conducted in accordance with the local legislation and institutional requirements. Written informed consent for participation in this study was provided by the participants or the participants’ legal guardians/next of kin.

## Author contributions

CZ: Conceptualization, Data curation, Formal analysis, Funding acquisition, Investigation, Methodology, Project administration, Resources, Software, Supervision, Validation, Visualization, Writing – original draft, Writing – review & editing. DL: Data curation, Formal analysis, Investigation, Resources, Software, Writing – review & editing. YW: Data curation, Formal analysis, Investigation, Methodology, Resources, Supervision, Validation, Writing – review & editing. LW: Investigation, Resources, Writing – review & editing. YH: Conceptualization, Data curation, Formal analysis, Investigation, Methodology, Software, Validation, Visualization, Writing – review & editing. JY: Formal analysis, Investigation, Resources, Supervision, Validation, Writing – review & editing.

## Footnotes

^1^https://www.fda.gov/

^2^https://cge.cbs.dtu.dk/services/ResFinder/

^3^http://www.mgc.ac.cn/VFs/main.htm

^4^https://pubmlst.org/organisms/acinetobacter-baumannii/

^5^https://itol.embl.de/upload.cgi
