# Supplementary material for: Risk factors and genetic characteristics of the carriage of hypervirulent and carbapenem-resistant Acinetobacter baumannii among pregnant women
Source: Front Microbiol. 2024 Mar 20;15:1351722. doi: 10.3389/fmicb.2024.1351722 (PMC10987950; doi:10.3389/fmicb.2024.1351722)
Supplement: Supplementary file 4 [file Data_Sheet_1.pdf]

Supplementary Material

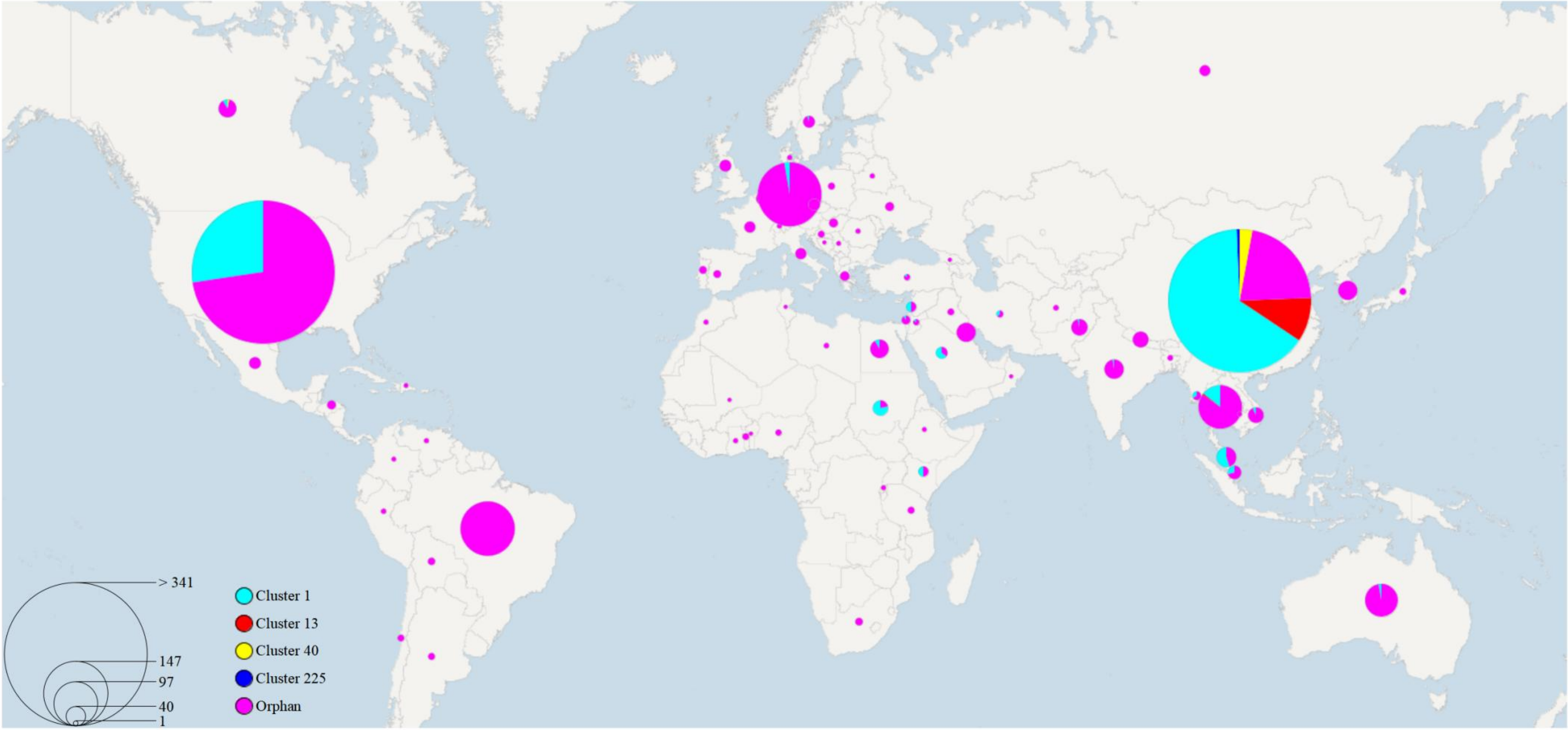

Supplementary Figure 1. Geographic distribution of the 4 maternity-related Clusters[RS] and orphans in the world. Circle size is proportional to the number of isolates. The map was created using Ridom SeqSphere+ (version 5.1.0).

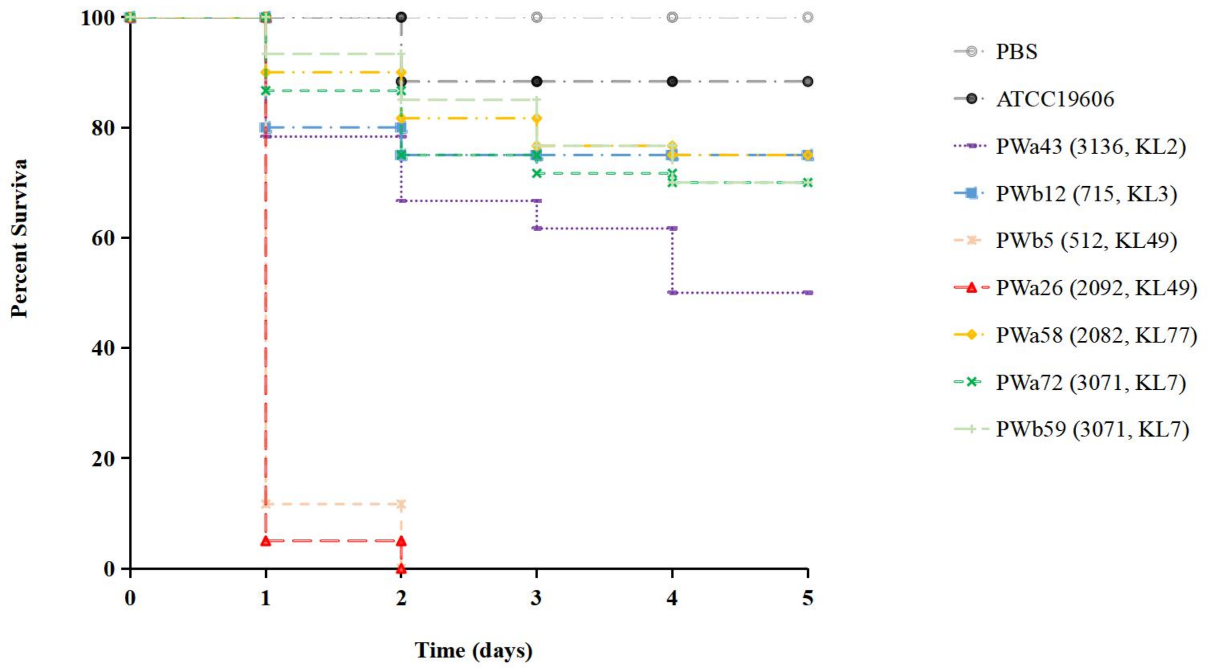

Supplementary Figure 2. Virulence potential of *Acinetobacter baumannii* isolates in a *Galleria mellonella* infection model. The effects of 1×10^6 colony-forming units of each *A. baumannii* isolate on survival were assessed in *G. mellonella*. Strain ATCC19606 and PBS were controls. At 120 hours (5 days), the survival rate of KL2 isolate PWa43 was 50%; the survival rates of KL3 isolate PWb12 and KL77 isolate PWa58 were both 75%; and the survival rates of the two KL7 isolates PWa72 and PWb59 were both 70%. The 2 KL49 isolates PWb5 and PWa26 exhibited 0% survival rates by two days post-infection, which were statistically significant differences from the others ($p < 0.001$ by log-rank test).

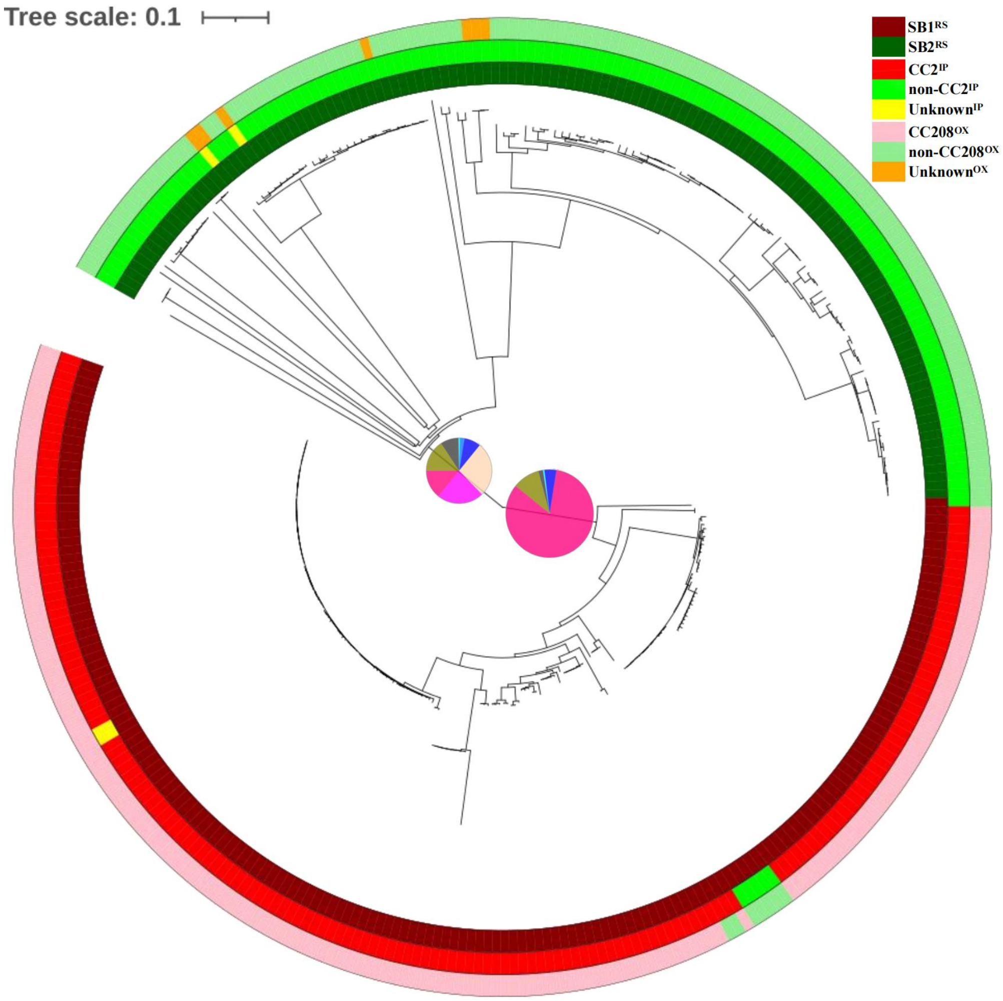

Geographic region		Cluster^{RS} (n)						
AFR	GH (n = 1)	Orphan						
	LY (n = 1)	Orphan						
CAM	HN (n = 3)	591 (2)	Orphan					
EUR	CZ (n = 2)	Orphan						
	FR (n = 2)	243						
	DE (n = 4)	243 (1)	Orphan					
	HU (n = 1)	Orphan						
	RU (n = 4)	494 (2)	774					
	SE (n = 2)	Orphan						
FEA	KH (n = 1)	Orphan						
	CN (n = 90)	13 (76)	193 (6)	621 (2)	Orphan (3)	Unknown		
	SG (n = 2)	Orphan						
	KR (n = 13)	418 (3)	537 (2)	590 (2)	741 (2)	Orphan		
	TH (n = 17)	13 (1)	223 (5)	369 (3)	402 (3)	Orphan		
	VN (n = 46)	52 (19)	167 (6)	257 (4)	287 (4)	588 (2)	Orphan	
MECA	GE (n = 1)	243						
	IN (n = 11)	156 (7)	760 (2)	Orphan				
	IL (n = 1)	Orphan						
	LB (n = 1)	Orphan						
	NP (n = 4)	592 (2)	Orphan					
	PK (n = 1)	Orphan						
NAM	CA (n = 7)	318 (4)	Orphan					
	US (n = 32)	100 (10)	136 (8)	428 (3)	534 (2)	Orphan		
OCE	AU (n = 36)	168 (6)	176 (6)	358 (3)	430 (3)	764 (2)	850 (1)	Orphan
SAM	BO (n = 3)	322						
	BR (n = 27)	112 (9)	386 (3)	Orphan (11)	Unknown			
	CL (n = 1)	Orphan						

Supplementary Figure 3. Phylogenetic tree constructed on the cgMLST of 314 KL49 *Acinetobacter baumannii* isolates from 27 countries in Africa (AFR), Central America (CAM), Europe (EUR), Far-East Asia (FEA), the Middle-East, and Central Asia (MECA), North America (NAM), Oceania (OCE), and South America (SAM). STRUCTURE software (version 2.3) was used to confirm the inferences using an admixture model, which can deal with complexities of data considering that individuals with mixed ancestry may have inherited part of their

genome from ancestors in population K. Posterior estimates for the parameters of interest were computed using a Markov chain Monte Carlo (MCMC) algorithm in ten parallel chains with a burn-in of 100,000 iterations and a run length of 10^6. The Evanno method was used to calculate the delta K in the program STRUCTURE HARVESTER (Evanno, et al., 2005; Earl and vonHoldt, 2012). To guarantee the optimum clustering, medians were calculated from 10 replicates for K using the FullSearch algorithm implemented in CLUMPP software (version 1.1.2) (Jakobsson and Rosenberg, 2007), and a cutoff of 0.7 was fixed for the clustering of isolates. Circle 1, as the outermost circle, depicts a Bayesian population structure analysis of 314 KL49 *A. baumannii* isolates using 2390-loci cgMLST. The figure shows the STRUCTURE ancestry coefficient (Q-matrix) effectively dividing the KL49 population into two groups, named $SB1^{RS}$ and $SB2^{RS}$. Circle 2, as the middle circle, depicts the corresponding CC2, non-CC2, and Unknown groups determined using the Pasteur MLST scheme. Circle 3, as the inner circle, depicts the corresponding CC208, non-CC208, and Unknown groups determined using the Oxford MLST scheme. The three methods of definition all enable the isolates to be divided into two distinct clades, indicating almost perfect agreement assessed using Cohen's weighted kappa coefficients ($\kappa_w > 0.9$). $SB1^{RS}$ isolates, corresponding to CC2/CC208, were predominant in FEA, representing 83.33% (150/180) of isolates. As opposed to the region-specific $SB1^{RS}$ clade, $SB2^{RS}$ isolates, corresponding to non-CC2/non-CC208, were broadly distributed, being present throughout much of the world. $SB2^{RS}$ represented 26.87% (36/134) of isolates in OCE, 23.13% (31/134) of isolates in SAM, 15.67% (21/134) of isolates in NAM, 14.18% (19/134) of isolates in FEA, 8.96% (12/134) of isolates in EUR, 8.21% (11/134) of isolates in MECA, 2.24% (3/134) of isolates in CAM, 0.75% (1/134) of isolates in AFR. The cluster (or orphan and unknown groups) in red in the table on the right indicates that the group determined using cgMLST by Ridom SeqSphere+ belonged to $SB1^{RS}$, $CC2^{IP}$, and $CC208^{OX}$. The cluster (or orphan and unknown groups) in green in the table on the right indicates that the group belonged to $SB2^{RS}$, non-$CC2^{IP}$, and non-$CC208^{OX}$. The orphan or unknown type determined using cgMLST in black in the table on the right indicates the definitions were not agreed upon in at least two of three schemes.

References

Earl, D. A., and vonHoldt, B. M. (2012). STRUCTURE HARVESTER: a website and program for visualizing STRUCTURE output and implementing the evanno method. *Conserv. Genet. Resour.* 4, 359-361. doi: 10.1007/s12686-011-9548-7

Evanno, G., Regnaut, S., and Goudet, J. (2005). Detecting the number of clusters of individuals using the software STRUCTURE: a simulation study. *Mol. Ecol.* 14, 2611-2620. doi: 10.1111/j.1365-294X.2005.02553.x

Jakobsson, M., and Rosenberg, N. A. (2007). CLUMPP: a cluster matching and permutation program for dealing with label switching and multimodality in analysis of population structure. *Bioinformatics* 23, 1801-1806. doi: 10.1093/bioinformatics/btm233

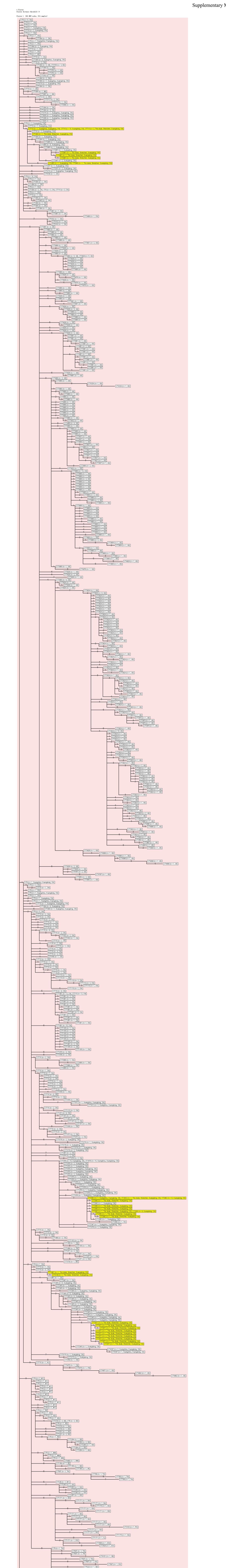

Supplementary Figure 4. Dendrogram of 974 Cluster[RS] 1 *A. baumannii* isolates based on cgMLST. Numbers on the connecting lines and the length of the lines represent the numbers of different alleles among the 2390 genes between linked isolates. The information in the box included the complex type, the number of corresponding isolates, the country of isolation, and the geographic location of isolates in Guangdong Province and its related cities (including Guangzhou and Shenzhen), China. The boxes associated with isolates from the present study are in yellow. MST, minimum spanning tree. AU, Australia; BE, Belgium; CA, Canada; CN, China; DE, Germany; EG, Egypt; IL, Israel; IN, India; IR, Iran; JO, Jordan; KE, Kenya; LB, Lebanon; MM, Myanmar; MY, Malaysia; PK, Pakistan; SA, Saudi Arabia; SD, Sudan; SE, Sweden; SG, Singapore; TH, Thailand; TR, Turkey; US, USA; VN, Vietnam.

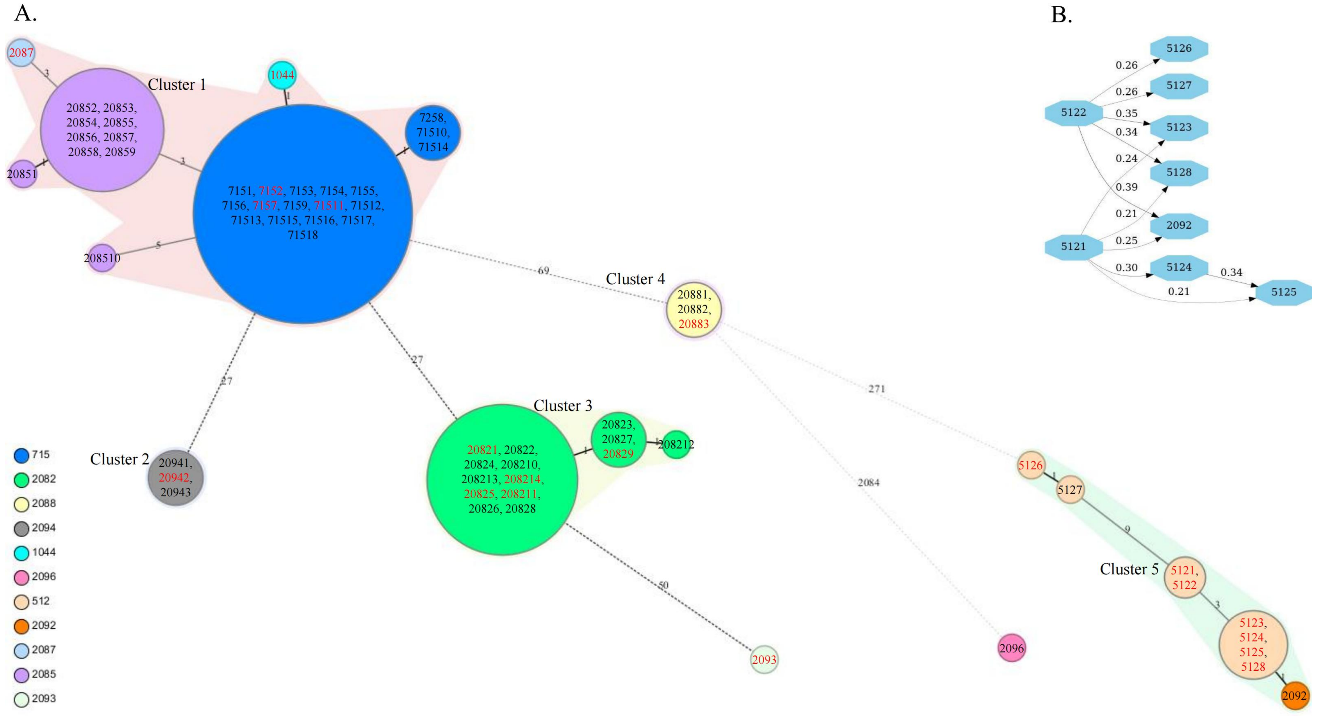

Supplementary Figure 5. The emergence and dissemination of KL49 carbapenem-resistant *A. baumannii* isolates in 2017 at Shenzhen People's Hospital, and the pathogenicity of recent high-risk CRAB carriage in pregnancy. **(A)** A minimum spanning tree (MST) of CRAB based on the cgMLST scheme using Ridom SeqSphere+. Each circle represents isolates belonging to a corresponding complex type (CT) based on the sequence analysis of 2390 cgMLST target genes. The codes in the circles refer to strain names; red strain names represent death cases. The numbers on the branches indicate distances in terms of the number of alleles. The cluster distance threshold is 9. Cluster 5 was the KL49 CRAB group. **(B)** SCOTTI (Structured Coalescent Transmission Tree Inference) reconstruction of between-patient transfer dynamics of the KL49 CRAB cluster. The isolates in the same Cluster 5 were selected to construct the transmission network. Core genome alignment was performed with the Harvest suite (version 1.2) (Treangen et al., 2014). The output file of core genome alignment was used to construct transmission trees with the BEAST2 (version 2.5.1) package SCOTTI (version 1.1.1), which combined genetic information from infection samples with epidemiological information of patient exposure to infection (De Maio et al., 2016). The chain was run for 100,000,000 steps, with parameter values recorded every 10,000 steps. The MCMC output was analyzed using Tracer (version 1.7.1) (Longair et al., 2011) to obtain the posterior distribution and the effective sample size (ESS) of all parameters (which were all above 1700) after a burn-in of 10%. Blue boxes represent infected patients, with corresponding strain names indicated within the box. Lines represent the predicted between-patient transfer events, and the numbers above the lines indicate the posterior probability of the transfer event. Between-patient transfer events were attributed to *A. baumannii* 5122 and 5121. An interesting observation emerging from this study was that a postpartum woman with the 5122 isolate might represent a super-spreader patient, a hotspot for the highly lethal strain colonization and transmission. The transmission events predicted by SCOTTI were not only direct transmission events between patients, but could also be indirect transmission events including unobserved and non-sampled intermediate colonized patients or environmental reservoirs in the hospital.

References

De Maio, N., Wu, C. H., Wilson, D. J. (2016). SCOTTI: efficient reconstruction of transmission within outbreaks with the structured coalescent. *PLoS Comput. Biol.* 12:e1005130. doi: 10.1371/journal.pcbi.1005130

Longair, M. H., Baker, D. A., and Armstrong, J. D. (2011). Simple neurite tracer: open source software for reconstruction, visualization and analysis of neuronal processes. *Bioinformatics* 27, 2453-2454. doi: 10.1093/bioinformatics/btr390

Treangen, T. J., Ondov, B. D., Koren, S., and Phillippy, A. M. (2014). The harvest suite for rapid core-genome alignment and visualization of thousands of intraspecific microbial genomes. *Genome Biol.* 15:524. doi: 10.1186/s13059-014-0524-x
